# Development of Apex Volar Deformity Following Volar Plating of Pediatric Radius Fractures

**DOI:** 10.2106/JBJS.OA.25.00317

**Published:** 2026-02-11

**Authors:** Rachel L. Lenhart, Pille-Riin Värk, Keith D. Baldwin, Jonathan G. Schoenecker, Christine M. Goodbody, Sulagna Sarkar, Apurva S. Shah

**Affiliations:** 1 Department of Orthopaedic Surgery, Medical College of Wisconsin, Milwaukee, Wisconsin; 2 Department of Pediatric Surgery, Tartu University Hospital, Tartu, Estonia; 3 Division of Orthopaedics, The Children's Hospital of Philadelphia, Philadelphia, Pennsylvania; 4 Department of Orthopaedics, Vanderbilt University Medical Center, Nashville, Tennessee

## Abstract

**Background:**

Plate fixation in skeletally immature children can cause angular deformity with longitudinal growth even when the plate does not overlie the adjacent physis. While this phenomenon has been described for the distal femur, angular deformity has not been reported following plating in other long bones. The aim of the study was to characterize whether sagittal-plane deformity occurs following volar plating of radius fractures in skeletally immature children and to determine associated risk factors.

**Methods:**

A retrospective review of volar plating of acute radius fractures in children with an open distal radius physis at a single institution was completed. In patients with at least 4 months of follow-up, the first radiograph and the last follow-up radiograph were evaluated for any change in sagittal angulation distal to the plate. Demographic information was obtained from the electronic medical record. Linear regression analysis was used to determine if distance from the plate to the physis, follow-up time, age, or coexisting ulnar fracture was predictive of any observed changes in angulation.

**Results:**

Sixty-one acute radius fractures treated with volar plating at a mean age of 12.1 years (67% male, 70.5% White, 90.2% non-Hispanic) were included. When analyzing by fracture location, 78% (21/27) of the distal-third radius fractures with appropriate follow-up developed at least 10° of apex volar angular deformity, with 44% (12/27) exhibiting greater than 20°. Middle-third and proximal-third fractures did not exhibit similar degrees of angulation (only 13% [4/30] and 0% [0/4] of included patients developed more than 10° of deformity, respectively). Linear regression analysis revealed distance of the plate to the physis and follow-up time to be strong predictors of angulation (both p < 0.0001).

**Conclusions:**

Children with radius fractures, particularly those in the distal-third, treated with a volar plate may develop apex volar angular deformity. While the exact rate of this phenomenon is unclear, these findings underscore the importance of strict surgical indications and vigilant postoperative monitoring beyond fracture healing, and represent a paradigm shift in understanding growth modulation following plating.

**Level of Evidence:**

Therapeutic Level IV. See Instructions for Authors for a complete description of levels of evidence.

## Introduction

Treatment of fractures in skeletally immature patients presents unique challenges. Juxta-physeal fracture can lead to bony deformity, even without direct involvement of the physis, such as in the classic Cozen phenomenon (valgus following proximal tibial fracture)^1,2^. Plate fixation can also induce similar growth disturbances^3^. A landmark study by Heyworth et al. created awareness of valgus deformity following lateral plating of distal diaphyseal femoral fractures^3^. However, reports of such deformities following plate fixation of radius fractures are notably infrequent^4^, and no dedicated investigation of this phenomenon has been performed.

This study aimed to evaluate pediatric radius fractures treated with volar plate fixation to determine whether angular deformity develops with longitudinal growth. The secondary aim was to identify risk factors for angulation. The hypothesis was that radius volar plating can cause apex volar deformity, with fixation closer to the physis increasing risk.

## Materials and Methods

Institutional Review Board approval was obtained to review surgically treated pediatric radius fractures between 2008 and 2022 at a tertiary care children's hospital. Current Procedural Terminology codes (25515, 25525, 25526, 25574, 25575, and 25607) identified patients from an institutional database. Records were reviewed to isolate skeletally immature children with volar plating. Exclusion criteria included partial or complete closure of the distal radial physis, alternate forms of fixation (e.g., Kirshner-wires and flexible intramedullary nails), dorsal plating, surgery more than 6 weeks after injury, or insufficient radiographic follow-up (i.e., less than 4 months). Fracture location, coexisting ulnar fracture, and demographics, including age at surgery, were recorded from the electronic medical record.

**Fig. 1:**
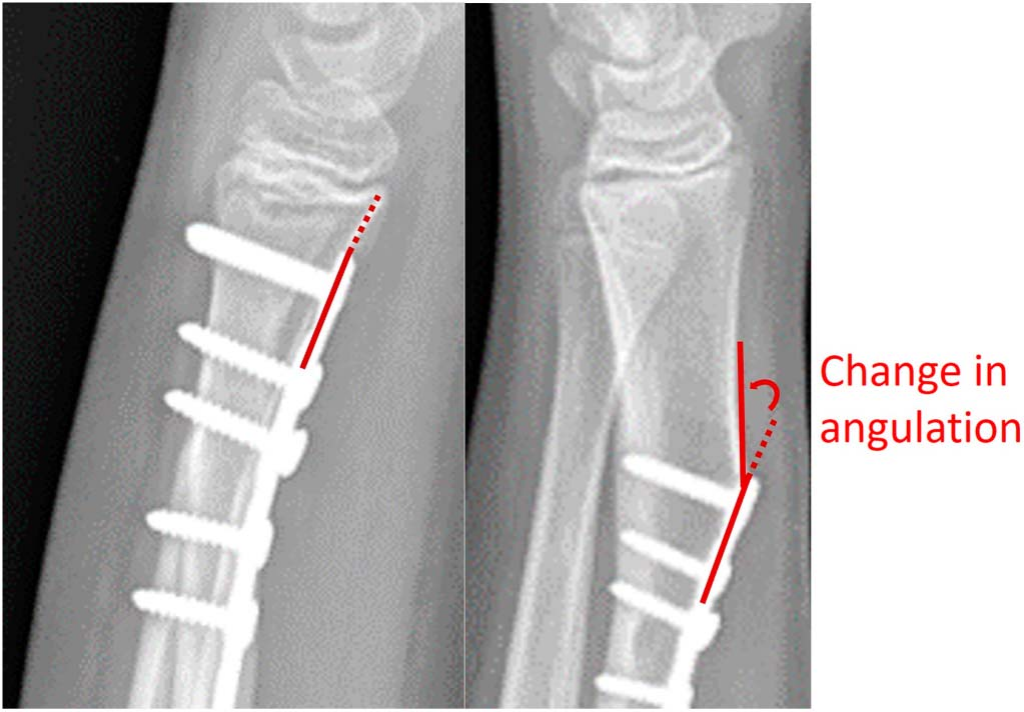
Lateral radiograph of the wrist demonstrating how angular deformity in the radius was measured.

The first postoperative radiograph and radiograph at most recent follow-up were analyzed. The primary outcome measure was change in sagittal angle of the radius just distal to the plate. Specifically, the angle between the plate and the volar radial cortex just distal to the plate was measured and compared between lateral radiographs (Fig. 1). In rare cases of excessive callus formation, the angle was measured down the axis of the bone just distal to the plate (rather than include the callus formation which did not represent true deformity). The distance between the plate and the physis on the first postoperative radiograph was recorded as a potential predictor variable. Owing to differences in radiographic technique, 10° was chosen as the cutoff to represent true deformity vs. measurement error.

Multiple linear regression analysis was used to determine a relationship between development of this deformity and patient age, distance from the distal aspect of the plate to the physis, coexisting ulnar fracture, and time to final follow-up. A simple analysis was performed initially without interaction terms. A second analysis was then completed including pairwise interactions and used stepwise analysis to remove terms that were not significant. Statistical analysis was completed using MATLAB (Version R2022b, Mathworks).

## Results

**Fig. 2:**
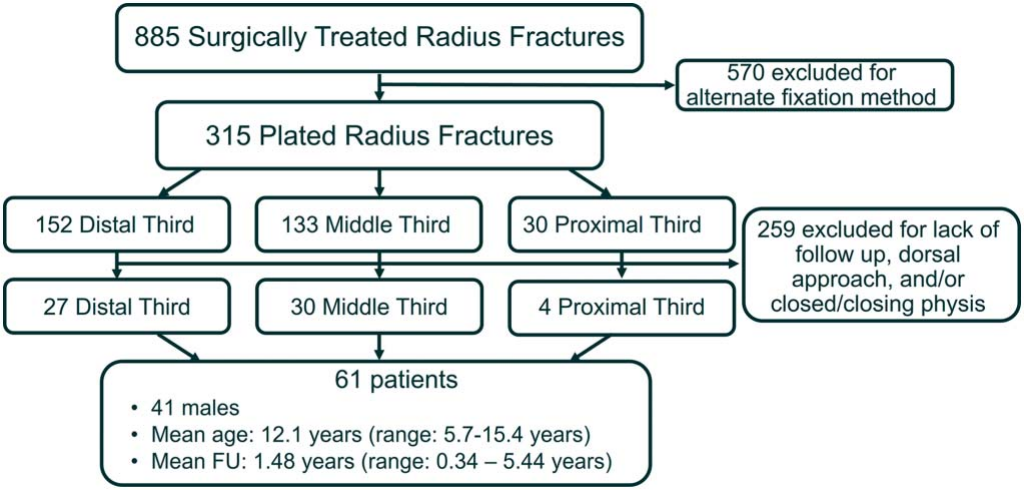
Flowsheet of excluded and included patients.

A total of 885 patients were extracted from the query, but most had alternative fixation methods. Three hundred fifteen patients had plated radius fractures. However, only 61 met inclusion/exclusion criteria, most common reason for exclusion being insufficient follow-up time (Fig. 2). Included patients had an average age of 12.1 years (range 5.7-15.4), 41 (67%) were male, 43 (71%) were White, and 55 (90%) were non-Hispanic. Mean radiographic follow-up was 1.5 years (0.3-5.4). The final cohort consisted of 27 distal-third, 30 middle-third, and 4 proximal-third fractures.

**Fig. 3:**
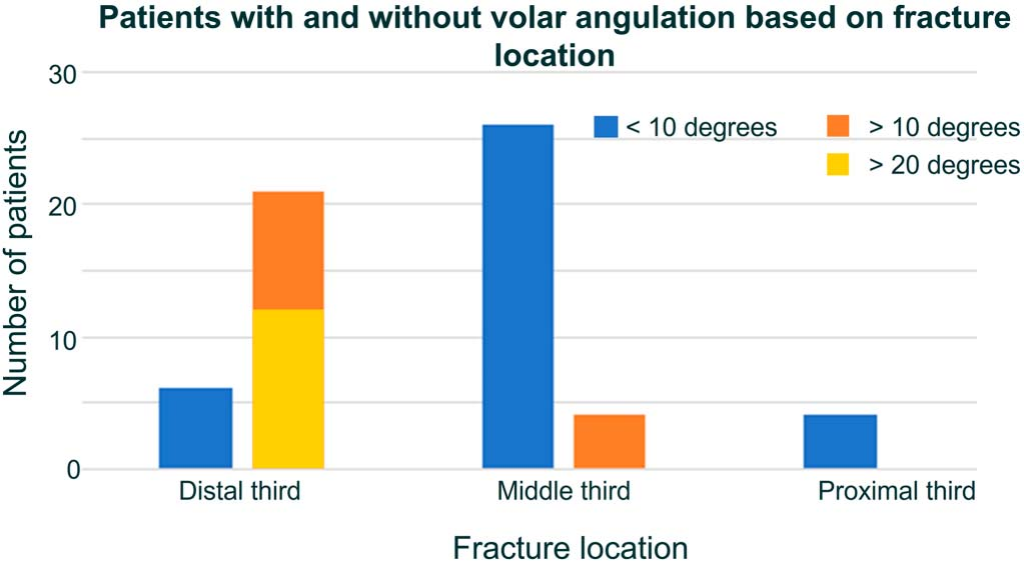
Number of patients with angular deformity after volar plating of radius fractures, stratified by fracture location.

Of the distal-third radius fractures, 21 (78%) developed apex volar deformity of greater than 10° and 12 (44%) had deformity greater than 20° (Fig. 3). Deformity was less common in other fracture locations with only 4 (13%) of the middle-third and 0 proximal-third fractures having greater than 10° of angulation. None with middle-third or proximal-third fractures demonstrated more than 20° of deformity. In the distal-third group, the mean angulation was 20.8° (range 3.0-49.4). Thirteen (48%) in the distal-third group had hardware removal, with 10 documented as symptomatic.

Multiple linear regression analysis revealed that all factors tested (age, coexisting ulnar fracture, follow-up time, and distance from plate to physis) were all significant predictors of developing angulation. The overall model fit the data well with R^2^ = 0.60 (p < 0.001, Table I). Initial plate distance to the physis and follow-up duration were highly predictive (both p < 0.001), with closer distance and longer follow-up predicting angulation. Coexisting ulnar fracture (p = 0.01) and age (p = 0.03) were also predictive, with an intact ulna and older age leading to more angulation (see Supplemental Table 1, and Supplemental Figure 1). When performing a stepwise linear regression model including interaction terms, the fit improved (R^2^ = 0.71, p < 0.001, see Supplemental Table II). The interaction terms between follow-up time and both distance to the physis (p < 0.01) and coexisting ulnar fracture (p < 0.001) drove the improved fit of the model, suggesting follow-up modulates the response to these variables. For example, for a given plate distance to the physis, follow-up time has a greater impact on predicted angulation in the model that includes interaction terms compared with the simple model (Table II).

**TABLE I:** Linear Regression Model Details for Simple Linear Regression (Without Interactions)

	Coefficients	Standard Error	*t* Stat	p-value
Intercept	11.74	5.86	2.00	0.05
Age at surgery	1.038	0.47	2.19	0.03
Coexisting ulnar fracture	−8.61	3.37	−2.55	0.01
Initial distance from plate to physis	−0.20	0.03	−6.79	<0.001
Follow-up time	3.36	0.80	4.22	<0.001

R^2^ = 0.60, Adjusted R^2^ = 0.57, p-value ≤0.001.

**TABLE II:** Results of Stepwise Linear Regression Including Interaction Terms

	Coefficients	Standard Error	*t* Stat	p-value
Intercept	2.42	5.00	0.48	0.63
Initial distance from plate to physis	−0.07	0.04	−1.92	0.06
Follow-up time	16.99	3.00	5.66	<0.01
Coexisting ulnar fracture	8.37	4.50	1.86	0.07
Initial plate distance × follow-up	−0.06	0.02	−3.38	0.001
Follow-up × coexisting ulnar fracture	−11.51	2.96	−3.88	<0.001

R^2^ = 0.71, Adjusted R^2^ = 0.68, p-value ≤0.01. Model was represented by 2 interaction terms (initial plate distance × follow-up and follow-up × coexisting ulnar fracture).

**Fig. 4:**
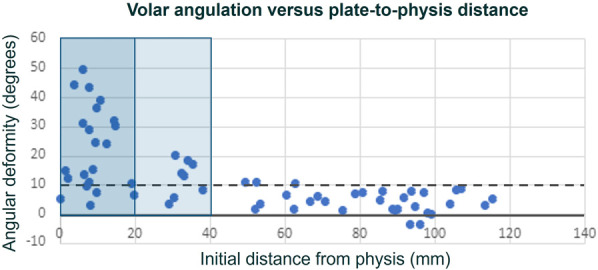
Relationship between distance to the physis and change in angulation after fracture fixation. The dashed line at 10° represents our estimate of the smallest measurable difference. Most changes in angle occur when the plate begins less than 4 cm from the physis, with the largest changes happening when the plate is less than 2 cm from the physis.

The relationship between the plate distance to the physis and development of angular deformity is represented in Figure 4. Notably, if the plate is within 2 cm of the physis, the patient has 42.9 times the odds of having angulation greater than 20° (95% confidence interval [CI] [4.9-372.7], p = 0.0007) and 17.0 times the odds of having angulation greater than 10° (95% CI [4.5-64.7], p < 0.0001).

## Discussion

This study aimed to determine if radius fractures in skeletally immature children treated with volar plating were at risk for apex volar deformity. Findings revealed that plates for distal-third fractures are particularly vulnerable, with 78% of distal-third fractures included in this study developing greater than 10° angulation. This previously unexplored phenomenon represents a need to shift the clinical paradigm and monitor patients beyond fracture healing.

Treatment of distal radius fractures in children is primarily dominated by closed reduction with or without percutaneous pinning^5^. Limited research exists regarding volar plating of distal-third radius fractures in skeletally immature patients. Prior studies found no functional differences compared with other fixation techniques, but did not longitudinally follow radiographs beyond healing^6,7^. Slightly more attention has been given to more proximal fractures, with near equivalence found between plates and intramedullary nails with regard to complications and functional outcomes^8^. Still, longitudinal assessment of bony morphology has been limited^9^.

**Fig. 5:**
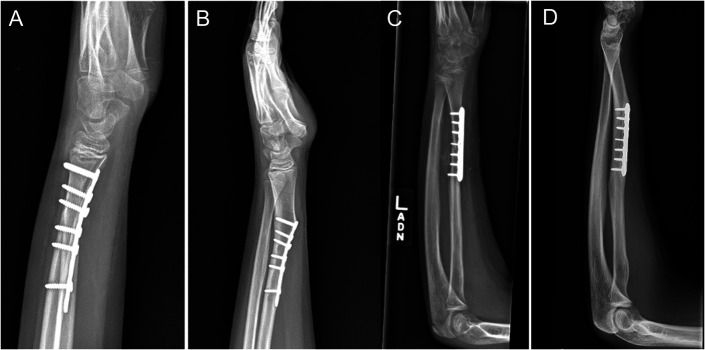
Two case examples of apex volar angulation after distal radius volar plating. Distal fracture in a 12-year-old boy 1 month after surgery (**Fig. 5-A**) and 2.8 years after surgery (**Fig. 5-B**). The right 2 images show the patient with fracture and plate further from physis (11-year-old boy, **Fig. 5-C** is 10 days after surgery, **Fig. 5-D** is 3.3 years after surgery).

While infrequent, deformity after radius volar plating has been reported previously. In a 2005 case report, a patient developed apex volar angulation volar plating of a fracture at age 12^4^. Of note, a dismissive letter to the editor followed^10^. Another recent case report focused on the plate's proximal migration, but inspection shows evidence of deformity distal to the plate^11^. Our study suggests this may be a common finding if plated distal-third fractures have additional follow-up (Fig. 5). In our cohort of patients with more than 4-month radiographic follow-up, a majority of distal-third fractures experienced more than 10° apex volar angulation. While the incidence here is likely biased, this previously unappreciated phenomenon should be considered when caring for these children. Anecdotally, surgeons seemed quite perplexed when encountering this secondary deformity, particularly when of larger magnitude. In one case, an magnetic resonance imaging was obtained looking for dorsal-sided growth arrest, which did not exist (see Supplemental Figure 2).

Deformity after plating of fractures is not a new concept. Plating of pediatric femoral shaft fractures can result in valgus deformity, particularly if the plate is within 2 cm of the physis^3^. Our study has similar findings, with plates within 2 cm showing high rates of larger angulation (Fig. 4). This highlights the need for further systematic studies to determine more precise rates of angulation and consideration of plate removal in skeletally immature children.

While there are similarities between deformity development in the femur and radius, notably the distal radius deformity occurs in the opposite direction of the distal femur. In the radius, the deformity has an apex on the same side as the plate (i.e., volar), whereas for the femur, the apex is opposite the plate (i.e., medial). Therefore, our findings challenge the previous theory of growth impedance following plating^3^. While it is beyond the scope of this paper to determine the reasons for the phenomenon to be contrary to the distal femur, it is interesting to speculate. One theory is related to how the plating techniques affect the periosteum near the growth plate. During distal femur submuscular plating, the periosteum is left intact^12^, which may tether the growth plate, leading to femoral valgus^13^. Conversely, during distal radius plating, the bone often is stripped of periosteum. Hemisection of the periosteum can lead to overgrowth^14^, which theoretically could lead to the results seen here. While this theory seems plausible, there are other possibilities, including local biomechanical factors, stress/strain relationships in differently shaped bones, or differing physeal blood supplies. These theories warrant more thorough investigation to determine potential contribution.

While we have demonstrated angulation, it is unclear what amount is clinically relevant. Radioulnar mechanics can be altered by a malunion after distal radius fractures^15,16^. Altered bony alignment can lead to distal radioulnar joint (DRUJ) instability or rotation loss^16-19^. Biomechanical studies suggest loss of supination with apex volar angulation, worsening with magnitude^20^ as the fracture moves from the distal to middle third^21,22^. One recent study of pediatric distal radius volar plating did not find pronation/supination differences at 29-month follow-up^6^. However, 58% of patients had hardware removal at 6 to 8 months, and radiographs were not analyzed after 6 weeks postoperatively^6^. Indeed in our study, 13 of the 27 distal-third fractures had hardware removal, with 10 being symptomatic, which is of interest since the rate of symptomatic hardware in adult distal radius is notably lower^23-26^. Reports of motion were too inconsistent to report here. Case reports of distal radial plating in children with subsequent deformity have noted symptoms of DRUJ instability^4^ or limited supination^11^. We argue that there is theoretical evidence that angulation caused by radius plating, if large, could result in supination loss and DRUJ instability.

In addition to plate distance to the physis, follow-up time was highly predictive of angulation with longer follow-up times showing more angulation. This suggests that the insult may continue to affect the physis with continued growth. Over half of our patients (33/61), including 10 distal-third fractures, had less than 1-year follow-up. It is possible that short follow-up may have missed deformity in some children. Most patients (11 of 12) with >20° angulation, had 1.5 or more years of follow-up. This suggests a need for a shift in clinical care, advocating that continued follow-up is imperative.

Older age and an intact ulna may also be more likely to develop angulation. Older age likely represents patients closer to peak height velocity^27,28^, as older children were often excluded for a closing physis. All patients in this study with angulation >20° were between 11 and 14.2 years, which is similar to peak height velocity ages in girls and boys^29^. Coexisting ulnar fracture being protective is harder to understand. Metaphyseal radius fractures with an intact ulna have high risk for redisplacement after closed reduction^30-32^. Perhaps a similar mechanism contributes to the development of angulation after plating due to asymmetric growth.

The clinical implications for these findings are twofold. First, given the rates of deformity in plated distal-third radius fractures, the indications for using this technique should be strict, particularly in young patients. It is possible that the secondary deformity could negate the short-term benefits of fracture alignment. When possible, nonoperative or other surgical techniques (e.g., percutaneous pinning and flexible nailing) should be used. Second, when plating cannot be avoided, the plate should be kept as far from the physis as possible and radiographic follow-up should be at least 6 to 12 months. Consideration should be given to hardware removal if deformity is progressive.

There are several limitations of this study. First, this was a retrospective single institution study, with surgeon-determined indications for treatment. The incidence and odds of deformity development are likely biased due to large number of excluded patients. All follow-up was at surgeon discretion, with many discontinuing radiographs after bony healing at 6 weeks. Hence, the incidence of the deformity could be overreported herein. However, if all patients excluded for follow-up lacked deformity, rates of development of angulation over 10° would still be 21% in the distal-third group. Owing to lack of standardization of the radiographs, we were unable to determine effects on volar tilt of the radius. We were also unable to classify by bone age which may be a better predictor than chronological age. Other questions also remain, including if the radius remodels in response to this deformity and if removal of the plate halts or reverses deformity.

In conclusion, radius fractures treated with volar plating have risk for apex volar deformity, particularly if the fracture is in the distal-third and the plate extends distally. These findings underscore the importance of vigilant monitoring for at least 6 to 12 months in children undergoing volar plating, with consideration of plate removal if angulation occurs. Taken together, these findings necessitate further prospective investigations to determine a more accurate incidence of deformity and unravel biology behind this phenomenon.

## Appendix

Supporting material provided by the authors is posted with the online version of this article as a data supplement at jbjs.org (http://links.lww.com/JBJSOA/B97). This content was not copyedited or verified by JBJS.
